# The use of photogrammetric fossil models in palaeontology education

**DOI:** 10.1186/s12052-020-00140-w

**Published:** 2021-01-13

**Authors:** John A. Cunningham

**Affiliations:** School of Earth Sciences, University of Bristol, Bristol, UK

**Keywords:** Palaeontology, Digital visualization, Earth science education, Digital fossils, Photogrammetry

## Abstract

Photogrammetry allows overlapping photographs of fossils to be taken and converted into photo-realistic three-dimensional (3-D) digital models. These models offer potential advantages in teaching palaeontology: they are cheap to produce, can be easily shared and allow the study of rare and delicate specimens. Here I describe approaches for using photogrammetric models in the teaching and learning of palaeontology. Little is known about how students perceive these models and whether they find them valuable in their learning. To address this, first-year university students taught using both fossil specimens and digital models were surveyed about their experience through an anonymous online survey. Most students found that the digital models were easy to use, helped them understand anatomy and were more useful than studying photographs. However, most did not see the models as a substitute for studying real fossils and felt they could learn more from physical models. Digital models are a useful addition to palaeontological education that can supplement real fossils and allow palaeontological education to take place in circumstances where handling of specimens is not possible.

## Introduction

Drawing, describing and identifying fossil specimens are among the most fundamental skills in palaeontology. These are vital for studying the fossil record and understanding the evidence it provides of the evolution of life in deep time. Learning these skills forms an important component of most secondary school geology courses and introductory geoscience courses taught in universities. Students traditionally study fossil specimens from a teaching collection housed in their institution that has either been built up by the staff or bought from fossil collectors and is sometimes supplemented by casts or physical models of fossils. However, digital approaches are now also possible and may even be essential in the post-Covid world.

Technological advances in computer-aided visualization over the last two decades have led to a digital revolution in palaeontological research (Cunningham et al. 2014; Sutton et al. 2014, 2016). This has allowed digital models of fossils to be created using various scanning technologies and to be widely shared and studied (Davies et al. 2017). Photogrammetry is particularly relevant for education as it allows three-dimensional (3-D) photo-realistic digital models to be produced (Bates et al. 2010; Falkingham 2012). Photogrammetry involves overlapping photographs of a fossil being converted into a 3-D digital model that can be manipulated by the user, who can rotate the specimen and zoom in or out to examine features of interest.

There are several potential advantages of using photogrammetric digital fossils in education as they are cheap to produce and can be easily shared (Lautenschlager and Rücklin 2014; Rahman et al. 2012). The digital models allow students to study important extinct organisms that are known only from specimens that are too rare to be commonplace in collections or too delicate to handle. Each student in a large class can study the same specimen, which can help ensure fairness in assessments. Finally, they allow students to study specimens when the teaching collections are not available, such as when they are studying out of hours or away from the campus. The fact that they can be easily distributed to large numbers of students is important in the wake of the Covid-19 pandemic when fossil handling can sadly no longer be considered a low-risk activity. At the time of writing, we appear to be entering a so-called ‘new-normal’, under which socially distanced teaching is expected to be required to help prevent the continued spread of the virus for an unknown length of time ahead. Digital fossils could go some way to allowing palaeontology education to continue under these conditions.

Despite the potential advantages of using photogrammetric digital fossils in education, little is known about students’ experience of being taught using digital models and their perception of the effect on their learning. Studies of digital fossils specimens in the teaching and learning literature have tended to focus on 3-D printed specimens rather that direct study of the digital models themselves (e.g. Grant et al. (2016)). Here I describe approaches for incorporating digital fossils into teaching sessions and evaluate the student perception of these methods by analysing data from a survey of first-year university students who have been taught using both fossil specimens and digital models of fossils.

## Using digital fossils in palaeontological education

### Digitizing specimens

**Fig. 1:**
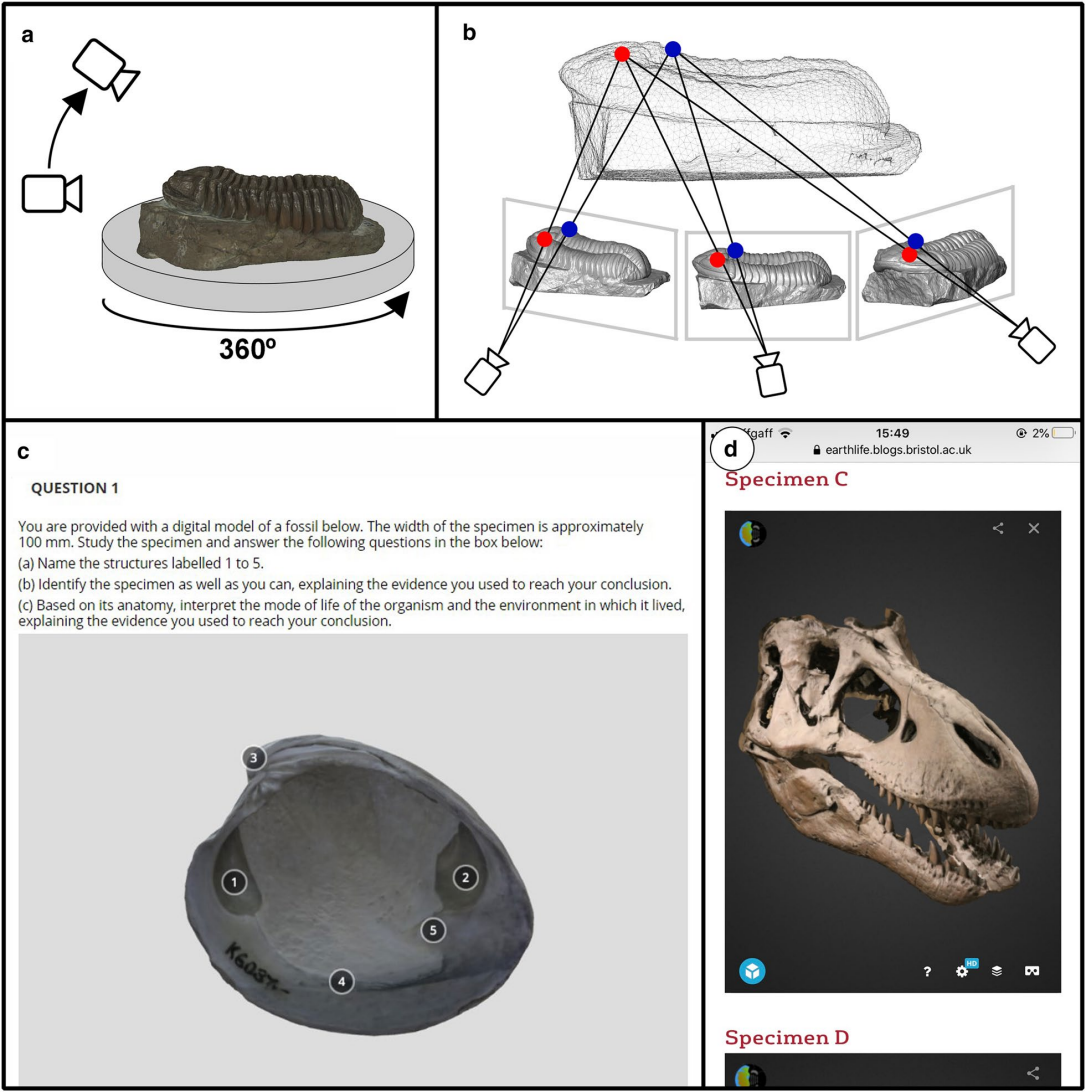
Schematic diagrams of photogrammetry methods (**a**, **b**) and examples of the use of photogrammetry in palaeontology education (**c**, **d**). **a** Photographs are taken of the specimen from multiple angles, often using a turntable for small specimens. **b** Photogrammetry uses triangulation to calculate the 3-D coordinates of points that occur in overlapping images; these are used to create a point cloud that defines the 3-D surface on which the colour and texture information from the photographs can be overlaid. The specimen in **a** and **b** is the trilobite *Calymene blumenbachii* (‘The Dudley Bug’) Lapworth Museum of Geology BIRUG BU53; the model was downloaded from the Lapworth Museum’s account on sketchfab.com and the reconstructions in **b** were made in the Avizo software. **c** An example of the use of a photogrammetry model in an exercise for students in the Blackboard VLE. The specimen is the bivalve *Mercenaria mercenaria* Paleontological Research Institution PRI 76728; the model was downloaded from the Digital Atlas of Ancient Life account on sketchfab.com and re-uploaded to the author’s account where the labels were added. **d** An example of a photogrammetry model that has been embedded into a virtual worksheet in a webpage and viewed on a smartphone. The specimen is a cast of the skull of *Tyrannosaurus rex* that is on display at the Museum of the Earth, Ithaca, NY and was embedded from the Digital Atlas of Ancient Life account on sketchfab.com

For simplicity, most educators will likely prefer to use the many high-quality models that have been freely shared (discussed in the next section), rather than creating models themselves. However, photogrammetric models of fossils can be created relatively easily using widely available equipment and software (Fig. 1). It may be desirable to create new models, for example, to allow students to study specimens in an institution’s own collection. All that is required is a digital camera and a computer with appropriate software; good results can be obtained within hours with photographs taken with a smartphone and processed using free software on a standard laptop computer (Otero et al. 2020). A detailed practical guide to photogrammetry is beyond the scope of this paper, but a summary of the technique – with suggestions for further reading – is given below.

For practical guides to capturing images for photogrammetry, the reader is referred to Falkingham (2012), Mallison and Wings (2014), Matthews et al. (2016) and Otero et al. (2020) as well as to the guide provided by the Digital Atlas of Ancient Life (https://www.digitalatlasofancientlife.org/methods-techniques/photogrammetry/). In summary, the specimen is photographed from multiple angles. For small specimens, a turntable and tripod are often used to help achieve consistent and high-quality images (Fig. 1a). Generally speaking, larger numbers of photographs produce higher quality models at the expense of longer processing time; optimal results are obtained when photographs overlap by approximately two-thirds, with each point on the specimen appearing in at least three different images. Photogrammetry works by using triangulation to calculate the 3-D coordinates of points that occur in overlapping images (Fig. 1b). The coordinates are used to create a point cloud that defines the 3-D surface and the colour and texture information from the photographs can be overlaid onto the point cloud (Sutton, et al. 2014). Once the photographs are captured the process of creating the 3-D model is usually automated in photogrammetry software, which locate matching points in different photographs and use them to generate a point cloud over which the textural information is overlain. A variety of free and commercial software are available. Reviews of free photogrammetry software and practical guides to using some of them in palaeontology are available at the website of researcher Peter Falkingham (www.peterfalkingham.com).

### Obtaining digital specimens from online collections

The simplest way for educators to include digital specimens in their teaching is to use models that have been shared online by their original creators. Many museums, institutions and individuals have made photogrammetric models of fossils freely available online; Table 1 lists some collections of fossil models that are likely to be useful for educators. The Paleontological Research Institution’s Digital Atlas of Ancient Life is particularly noteworthy, containing over 500 fossil models. The proprietary website Sketchfab (www.sketchfab.com) hosts a large number of freely available photogrammetric fossil specimens including those from the Digital Atlas of Ancient Life and several other museum collections (see Table 1), as well as models that can be bought. It is straightforward to incorporate models from Sketchfab into teaching materials as discussed below.

**Table 1:** A selection of useful sources of photogrammetry models for palaeontology education

Collection	Website(s)	Comments
Digital Atlas of Ancient Life	www.digitalatlasofancientlife.org/vc/ www.sketchfab.com/DigitalAtlasOfAncientLife	Over 500 specimens mainly from the Paleontological Research Institution collections; test specimens available for educators
GB3D Type fossils	www.3d-fossils.ac.uk/ www.sketchfab.com/3dFossils	Fossil type specimens from the UK
Smithsonian Institution	www.3d.si.edu/collections/hominin-fossils	Fossil hominins, mainly crania
Natural History Museum, London	www.sketchfab.com/NHM_Imaging	Fossil and extant specimens from the museum collections
Lapworth Museum, Birmingham	www.sketchfab.com/LapworthMuseum	Fossil and other geological specimens from the museum collections
Société Géologique de France	www.sketchfab.com/sgfrance	Fossils and other geological specimens
African Fossils	www.africanfossils.org	Hominid and other vertebrate fossils mainly from the Cenozoic of Lake Turkana
MorphoSource	www.morphosource.org	3-D datasets of fossil and extant taxa including photogrammetry models; a list of specimens for educators is provided
MorphoBank	www.morphobank.org	Morphological datasets including photogrammetry models
MorphoMuseuM	www.morphomuseum.com	3-D datasets of fossil and extant vertebrates including photogrammetry models

### Creating virtual worksheets

Instructors may wish to embed digital fossils into virtual worksheets for each teaching session or assessment. These worksheets can include both the models and the instructions for exercises that students are to complete. They can be designed so that the instructor can set exercises in the same way as they do with physical specimens. Exercises might test the ability to identify, draw and label fossils, but can also involve more complex exercises that build on fossil identifications using the information in tasks developing skills in areas such as stratigraphic, palaeoecological or palaeobiogeographical analysis. Using virtual worksheets has the advantages of making the activities clear for the student and preventing the need to click on multiple links to complete an exercise. They can also include additional important information about the specimens. For example, as many models lack a scale bar, it is good practice to include this information in the virtual worksheet. There are two main ways to create virtual worksheets that include 3-D models: as a PDF file, or on a webpage.

Photogrammetric models can be embedded into the PDF file format and manipulated by the user using Adobe’s freely-available and widely-used Acrobat Reader software (Lautenschlager 2014; Lautenschlager and Rücklin 2014). Using PDF files to create worksheets has the advantage that the file can be downloaded, and the exercise can be completed offline. In addition, the metadata associated with the model is not visible to the student: this is useful for exercises requiring taxonomic identifications where the metadata may reveal the answer. To create 3-D PDF files, the photogrammetric model must first be converted to the U3D format; this process is described in detail by Lautenschlager (2014).

A more straightforward way to incorporate photogrammetric models into virtual worksheets is to embed models obtained from www.sketchfab.com into webpages that can be viewed on standard computers, smartphones or tablets (Fig. 1c, d). To do this, the instructor can choose the ‘embed’ option under the model on the Sketchfab website and then copy the HTML code that is provided into the host webpage. The code can be embedded easily into any webpage including common Virtual Learning Environments (VLEs) such as Blackboard and Moodle (Fig. 1c). Embedding models in the VLE used by the institution allows the student to study on a familiar platform and enables the instructor to monitor student responses. To embed a model into either Blackboard or Moodle the instructor can create an item or test question and then select the HTML option in the toolbar above the text editor and paste in the embed code.

A potential disadvantage of directly embedding models from Sketchfab is that the title of the model will appear on the host website (as will a link to the model on the Sketchfab website where there may be a description of the model). These are likely to include information such as the name of the taxon, which will limit the kinds of questions that can be asked without the student being able to look up the answer easily. However, where the owner and license allow it, it is simple for an educator to download a model using the glTF format and then upload it to their own Sketchfab account without the identifying metadata. The Digital Atlas of Ancient Life have released models without any additional information for use by educators in assessment.

When models have been uploaded to a Sketchfab account, annotations can be added. These appear as numbers on the specimen but can be linked to a longer description that becomes visible when the student clicks on the number. These can either be used to illustrate the various features of a fossil specimen or, if the descriptions are removed, to test the students’ knowledge. Figure 1c shows an example of a test question where students are required to name annotated structures and identify a fossil specimen.

## Evaluation

Students taking ‘Evolution of Earth and Life’ – a five-week subunit of an introductory Geology course in a research-intensive UK university – were surveyed after being taught using both fossil specimens and photogrammetric digital fossils. The practical component of this subunit comprises five sessions, each of which focuses on identifying and interpreting the mode of life of fossils from a major group of animals. The first three sessions were taught using fossil specimens and casts from the institution’s teaching collection. The final two sessions were taught entirely online due to the Covid-19 pandemic. These sessions were taught using a combination of photographs and 3-D photogrammetric models. The digital models were obtained from www.sketchfab.com and were embedded into online worksheets created in a WordPress webpage containing questions for the students to answer. The students could obtain help and feedback from staff or postgraduate demonstrators during the practical sessions, either through a discussion board or by live audio or video link.

The week after completing the final teaching session, students were emailed a link to an anonymous online questionnaire designed to assess their perception of learning from the photogrammetric models. The design was chosen to gain insight into how students perceive the use of the models and to compare between different methods for delivering training in fossil anatomy. The students were asked to what extent they agreed with five statements about the digital specimens: (1) The 3-D digital fossils were easy to use; (2) The 3-D digital fossil helped me to understand fossil anatomy; (3) 3-D digital fossils can replace real fossil specimens; (4) I learned more from the 3-D digital fossils than I could from photographs; and (5) I could learn more from physical models of fossils than from the 3-D digital fossils. Each of the questions had five options: strongly disagree, somewhat disagree, neither agree nor disagree, somewhat agree, and strongly agree.

## Results

**Fig. 2:**
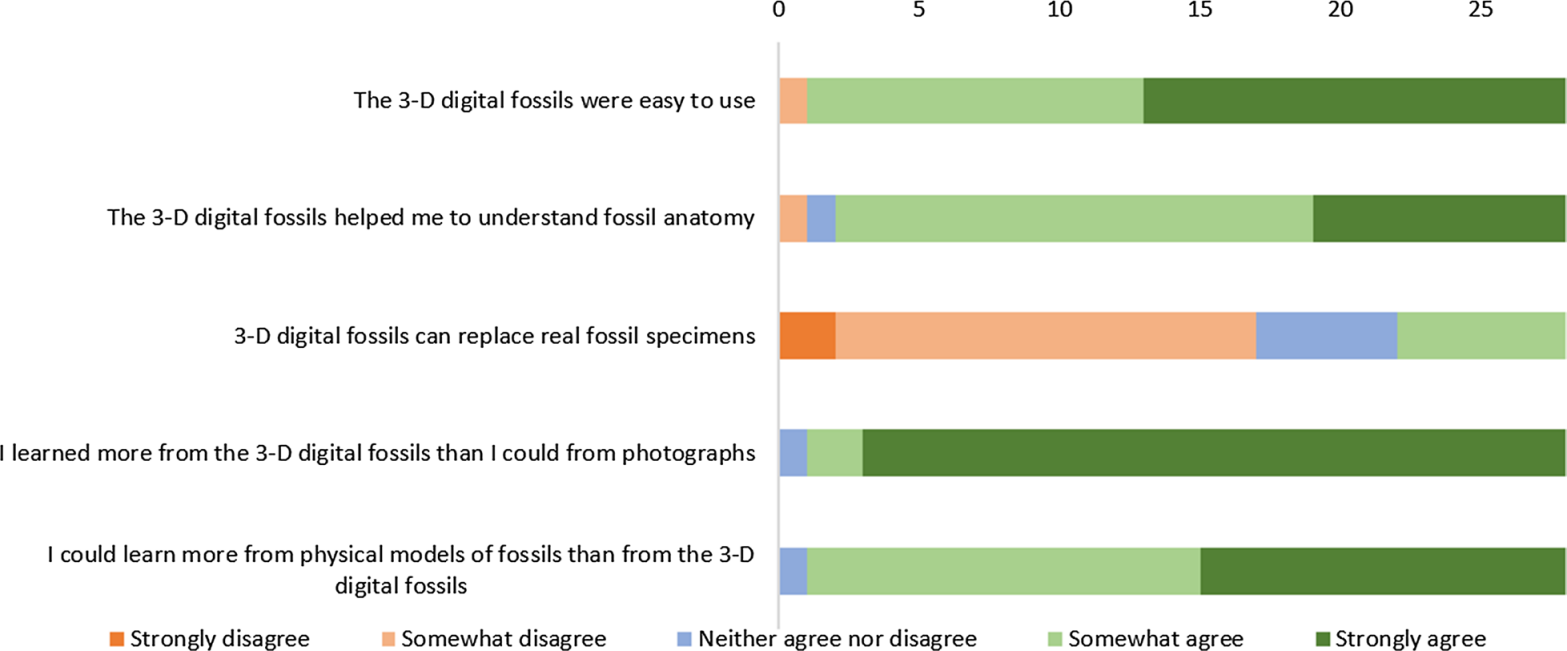
Summary of student responses to the survey questions about learning from digital fossil specimens. The axis shows the number of students giving each of the responses

The questionnaire was completed by 28 students, which represents 36% of the 77 students registered on the unit. All 28 students gave responses to all five statements and 15 answered the free text question. The numbers of students agreeing with each statement are summarized in Fig. 2. The full results of the survey are provided in Additional file 1.

Of the 28 students, 27 either somewhat agreed (12 students) or strongly agreed (15 students) that the digital models were easy to use. This was reflected in free-text comments, with students noting, for example, that ‘they’re very easy to use, they load quickly and maintain the quality of the image which is useful given they’re quite detailed images’. However, some students did note problems with using the models such as that they ‘need to be in higher resolution’ and that they were ‘a bit slow’ on a mobile phone. Others noted limitations in the way they were presented, such as the lack of scale information or the lack of an explanation that some features might not be visible on particular specimens. Nonetheless, all students bar two either somewhat agreed (17 students) or strongly agreed (9 students) with the statement that the models had helped them to understand fossil anatomy. Several students commented that they found the tool ‘useful’ for their learning.

All students apart from one felt that they could learn more from the digital fossils than from photographs of fossils, with 25 of the 28 students strongly agreeing. However, the same number of students either somewhat agreed (14 students) or strongly agreed (13 students) with the statement that they could learn more from physical models of specimens than they could from the digital fossils. Finally, the majority of students (17 of the 28 students) disagreed with the statement that digital fossils could replace the study of real fossil specimens (15 somewhat disagreed and 2 strongly disagreed). This was reflected in comments that the models ‘can't really fully replace the use of hand specimens’ and that the experience of handling the specimens could not be replicated. However, several made comments along the lines that ‘under the circumstances [i.e. the Covid-19 pandemic] these models are a good substitute’.

## Discussion

### Limitations of the study

The study is designed to assess student perception and so does not provide information on the value of photogrammetry models for learning or of their impact on student performance. The survey is further limited by both low participation (36%) and the fact that student feedback of teaching can be influenced by many factors that can limit its utility (Holland 2019). It is also possible that the preference of students could vary based on the tasks they are asked to complete. Research on the use of the same physical and digital kinematic simulations in teaching both geometry and robotics found that students in the two groups had different preferences: geometry students preferred the digital simulations while robotics students preferred the physical simulations (Pan et al. 2006).

### Strengths and weaknesses of photogrammetry models in palaeontology education

Given the potential advantages of digital fossils and the current need for socially distanced teaching and learning, it is reassuring to find that most students find digital fossils easy to use and more useful for their learning than studying photographs. Where physical specimen handling is not possible, the findings suggest that digital fossils may be preferable to photographs of specimens. The findings also highlight the potential of photogrammetry models for use in distance learning and in allowing students who cannot access regular classes a means of studying fossil material.

The limitations of digital models in teaching should also be considered carefully. Several students reported problems with slow loading or limited resolution. It seems likely that these issues mainly result from limitations of the students’ devices or internet bandwidth rather than with the models themselves, but in either case they are preventing some students from utilizing the models fully and need to be evaluated further. It is vital that instructors and institutions ensure that students have the necessary technology to access the teaching materials to avoid disadvantaging these students.

The survey results suggest that students prefer to study authentic fossil specimens where possible. There are major advantages of using original fossil specimens including the ability to appreciate subtle features that may be lost in other formats and providing students with the authentic experience of studying fossil material. Previous research has recognised the importance of “the real stuff” for drawing visitors to natural history museums (MacFadden 2008) and for engaging students and catalysing learning in outreach and education (Harnik and Ross 2004). The sense of wonder that many people encounter when handling the fossilized remains of an organism that lived millions of years ago can probably never be fully replicated.

The perception of students in this study that physical models of fossils are more beneficial to learning than digital models is borne out in the teaching and learning literature in anatomical sciences. For example, Saltarelli et al. (2014) found that anatomy students performed worse in assessments when trained using multimedia simulations rather than cadavers. Similarly, Preece et al. (2013) assessed the effect of learning from textbook illustrations, physical models and digital models on students’ ability to identify anatomical structures. They found that students using the physical models had significantly higher scores than those using either of the other two methods. These findings (along with the preference of students for physical models over digital models) suggest that, where conditions and budgets allow, physical models may be advantageous. 3-D printing can achieve this by allowing digital fossils to be converted to physical models, but with the loss of the colour information from the specimen surface (Rahman, et al. 2012). Grant et al. (2016) have shown that the use of 3-D printed fossil specimens can be effective in secondary education.

Despite the strengths of physical models, a strong case can be made for augmenting physical specimens and models with digital models. Findings from anatomical science suggest that students whose traditional teaching of anatomy from cadavers is supplemented with 3-D media perform better in assessments than those taught only through the traditional methods (Peterson and Mlynarczyk 2016). An approach that combines digital models with fossil specimens can exploit these educational advantages (and the general advantages of digital models of fossils discussed above) while retaining the benefits of handling fossil specimens.

## Conclusions

Studying fossil specimens is an essential part of a palaeontological education and digital models cannot completely replace this. However, students find digital fossils easy to use and believe they can learn more from them than from photographs. Digital fossil models can therefore play a key role in the teaching and learning of palaeontology. Situations where digital fossils are particularly useful include: socially distanced or remote teaching and learning; studying specimens known only from rare specimens or expensive models; providing study material when students are unable to access teaching collections; and providing identical material to all students in assessed exercises to ensure fairness. Photogrammetry models can be used to complement fossil specimens to take advantage of these benefits and they can allow palaeontological education to take place in circumstances where studying physical specimens is not possible.

## Supplementary Information

**Additional file 1:** **Tables S1 and S2.** Participants responses to the survey questions.

## Data Availability

All data and material generated or analysed in this study are available in Additional file 1.
