# Supplementary material for: The use of photogrammetric fossil models in palaeontology education
Source: Evolution (N Y). 2021 Jan 13;14(1):1. doi: 10.1186/s12052-020-00140-w (PMC7804905; doi:10.1186/s12052-020-00140-w)
Supplement: Supplementary file 1 — Additional file 1: Tables S1 and S2. Participants responses to the survey questions. [file 12052_2020_140_MOESM1_ESM.docx]

**Supporting Material**

**Supplementary Table 1**: Participants responses to the survey questions. Responses are coded numerically (1 for strongly disagree, 2 for somewhat disagree, 3 for neither agree nor disagree, 4 for somewhat agree and 5 for strongly agree). The questions were: (Q1) The 3-D digital fossils were easy to use; (Q2) The 3-D digital fossil helped me to understand fossil anatomy; (Q3) 3-D digital fossils can replace real fossil specimens; (Q4) I learned more from the 3-D digital fossils than I could from photographs; and (Q5) I could learn more from physical models of fossils than from the 3-D digital fossils.

| **Participant** | **Q1** | **Q2** | **Q3** | **Q4** | **Q5** |
| --- | --- | --- | --- | --- | --- |
| **P1** | 5 | 4 | 2 | 4 | 4 |
| **P2** | 5 | 4 | 2 | 5 | 4 |
| **P3** | 5 | 5 | 4 | 5 | 4 |
| **P4** | 5 | 5 | 4 | 5 | 4 |
| **P5** | 5 | 5 | 3 | 5 | 3 |
| **P6** | 5 | 4 | 2 | 5 | 4 |
| **P7** | 2 | 3 | 1 | 3 | 5 |
| **P8** | 5 | 4 | 2 | 5 | 4 |
| **P9** | 5 | 4 | 2 | 5 | 4 |
| **P10** | 4 | 2 | 1 | 5 | 5 |
| **P11** | 5 | 4 | 2 | 5 | 5 |
| **P12** | 4 | 4 | 3 | 5 | 5 |
| **P13** | 4 | 5 | 4 | 5 | 5 |
| **P14** | 4 | 4 | 2 | 5 | 5 |
| **P15** | 5 | 4 | 3 | 5 | 5 |
| **P16** | 4 | 5 | 4 | 5 | 4 |
| **P17** | 5 | 4 | 4 | 5 | 4 |
| **P18** | 4 | 5 | 3 | 5 | 4 |
| **P19** | 5 | 4 | 2 | 5 | 5 |
| **P20** | 5 | 4 | 2 | 4 | 4 |
| **P21** | 4 | 4 | 2 | 5 | 5 |
| **P22** | 4 | 5 | 2 | 5 | 5 |
| **P23** | 4 | 4 | 2 | 1 | 5 |
| **P24** | 4 | 4 | 2 | 5 | 4 |
| **P25** | 5 | 4 | 2 | 5 | 5 |
| **P26** | 4 | 4 | 2 | 5 | 4 |
| **P27** | 4 | 5 | 3 | 5 | 5 |
| **P28** | 5 | 5 | 4 | 5 | 4 |

**Supplementary Table 2:** Students’ responses to the free text question ‘Do you have any other comments about studying the 3D digital fossils?’

| **Participant** | **Comment** |
| --- | --- |
| **P1** | It is a valuable tool to use outside the practical rooms for revision. However, it can't really fully replace the use of hand specimens and samples |
| **P2** | N/a |
| **P3** |  |
| **P4** | A bit slow on phone but otherwise all good |
| **P5** |  |
| **P6** |  |
| **P7** | For 3D fossils to be useful, the model needs to be in higher resolution because blurring is quite problematic for me when identifying details of fossils |
| **P8** | i Don’t think 3d fossils will be able to full replace actual samples, as samples you get the feel of and they are a bit clearer. But under the circumstances these models are a good substitute, and are much better than photos. |
| **P9** | Sometimes it’s hard to see fine detail |
| **P10** |  |
| **P11** | I believe that some of the fossils we viewed did not show certain features that we were expected to study, but I feel that this could be overcome by having a number of samples for each specimen, as we would do in a laboratory. |
| **P12** |  |
| **P13** | The 3D model falls down in a few things. Being able to run your finger across the specimen in order to detect contours, the texture of the fossil and also the weight. Also the zooming function is jumpy and it is hard to get to the desired distance. |
| **P14** |  |
| **P15** |  |
| **P16** | The fossils were much better than I expected. I think it did show me personally that the practical stuff could actually probably be done remotely mostly. Obviously to be fully inept physical specimens are the best but it was interesting as a student |
| **P17** | Harder to study the texture online or looking at the fossil at different viewpoints that the cameras may not be able to access. |
| **P18** | 3D fossils were very useful, whilst working away from practical labs |
| **P19** | 3D models allowed for us to describe obvious structures and characteristics of the fossil easily but not really on small details such as less obvious parts or textures . |
| **P20** | A scale of size would be helpful. |
| **P21** |  |
| **P22** |  |
| **P23** |  |
| **P24** | I'd never used them before to understand fossils but I enjoyed it and found the tool useful. |
| **P25** | Maybe |
| **P26** |  |
| **P27** |  |
| **P28** | They're very easy to use, they load quickly and maintain the quality of the image which is useful given they're quite detailed images/high resolution. |

This comment was sent by email as it was too long for the online form:

“The insides of the fossils weren't visible or accurate, making some questions hard to answer. For example, when trying to view the insides/ inner areas of the fossils, it was either just a grey blur or a low res texture of what was being displayed on the outside of the shell. A different route to take for producing models could be finding a digital sculpting artist,  who could reproduce the fossils better, instead of what I assume was just a scan and texture map.”
